# RSero: A user-friendly R package to reconstruct pathogen circulation history from seroprevalence studies

**DOI:** 10.1371/journal.pcbi.1012777

**Published:** 2025-02-03

**Authors:** Nathanaël Hozé, Margarita Pons-Salort, C. Jessica E. Metcalf, Michael White, Henrik Salje, Simon Cauchemez

**Affiliations:** 1 Mathematical Modelling of Infectious Diseases Unit, Institut Pasteur, Université Paris Cité, U1332 INSERM, UMR2000 CNRS, Paris, France; 2 Université Paris Cité, INSERM, IAME, F-75018, Paris, France; 3 Institut Pasteur, Epidemiology and Modelling of Antimicrobials Evasion research Unit, Paris, France; 4 Université Paris-Saclay, UVSQ, Inserm, CESP, Anti-infective Evasion and Pharmacoepidemiology Research Team, Montigny-Le-Bretonneux, France; 5 MRC Centre for Global Infectious Disease Analysis, School of Public Health, Imperial College London, London, United Kingdom; 6 Department of Ecology and Evolutionary Biology, Princeton University, Princeton, New Jersey, United States of America; 7 Department of Global Health, Infectious Disease Epidemiology and Analytics G5 Unit, Institut Pasteur, Université Paris Cité, Paris, France; 8 Department of Genetics, University of Cambridge, Cambridge, United Kingdom; Northeastern University, UNITED STATES OF AMERICA

## Abstract

Population-based serological surveys are a key tool in epidemiology to characterize the level of population immunity and reconstruct the past circulation of pathogens. A variety of serocatalytic models have been developed to estimate the force of infection (FOI) (i.e., the rate at which susceptible individuals become infected) from age-stratified seroprevalence data. However, few tool currently exists to easily implement, combine, and compare these models. Here, we introduce an R package, *Rsero*, that implements a series of serocatalytic models and estimates the FOI from age-stratified seroprevalence data using Bayesian methods. The package also contains a series of features to perform model comparison and visualise model fit. We introduce new serocatalytic models of successive outbreaks and extend existing models of seroreversion to any transmission model. The different features of the package are illustrated with simulated and real-life data. We show we can identify the correct epidemiological scenario and recover model parameters in different epidemiological settings. We also show how the package can support serosurvey study design in a variety of epidemic situations. This package provides a standard framework to epidemiologists and modellers to study the dynamics of past pathogen circulation from cross-sectional serological survey data.

## Author summary

Antibodies are immune markers of past infections. Seroprevalence studies that characterize the seropositivity status in the population have been used for decades in epidemiological studies to assess epidemiological parameters such as population immunity and estimate the risk of future infections. In addition, when the age of participants is available, their seropositivity status can be used to reconstruct the history of circulation of the pathogen in the studied population. We developed Rsero, a R package that provides a pipeline of methods to store age-stratified serological datasets, analyze serological surveys, estimate the history of circulation of a pathogen and compare different transmission models. Statistical inference is done using Bayesian approaches. We introduce new models to detect past epidemics from serological surveys and we extend existing models of antibody decay to any transmission model. Rsero is easy to use and is designed for the broad community of epidemiologists and modellers that are working on serological studies.

## Introduction

Antigen-specific antibodies are immunological markers of past infection. By analyzing blood samples from a representative sample of the population, serological surveys can be used to characterize the distribution of these antibodies in a population. Such studies are of major importance for quantifying key epidemiological variables such as population immunity and population susceptibility to future epidemics, and can play a key role in ascertaining infection levels when surveillance systems only detect a fraction of infections, for example in the study of SARS-CoV-2 [1], yellow fever [2], Rift Valley fever [3] and dengue[4].

The use of serosurveys is not limited to the estimation of population immunity. When information about the age of individuals is available along with their serostatus, these data can also be used to reconstruct the history of pathogen circulation. For example, if a pathogen is characterized by low level endemic circulation with a force of infection (i.e., the per capita rate at which susceptible individuals are infected) of 2% per year, we expect that seroprevalence will slowly increase with age (e.g., 0%, 18%, 33% in those aged 0, 10 and 20 y.o., respectively). By analyzing how seroprevalence changes with age, serocatalytic models can reconstruct the historical force of infection. The simplest serocatalytic model that assumes a constant force of infection was introduced by Muench in 1934 [5].

Since Muench, many extensions of the serocatalytic model have been proposed to enhance the analysis of age-stratified serosurveys. For example, a couple of cross-sectional serosurveys can now be used to assess how the force of infection changed over the last few decades, allowing the identification of past epidemics that were previously missed because of a lack of adequate surveillance [6]. Serocatalytic models can also be used to test competing modes of circulation (e.g., endemic circulation vs irregular outbreaks) while accounting for the issue of cross-reactivity [7,8]; or to characterize changes in transmission following an intervention [9]. Many extensions of serocatalytic models have been proposed, for example, by relaxing the assumption of lifelong immunity to capture reinfections and seroreversion [10–12].

Despite their shared mechanistic foundation, few tools are available to allow epidemiologists and modellers to easily apply and move between different assumptions. The *serosolver* package enables analysis of influenza antibody landscapes [13]; and the *serosim* package facilitates simulation of vaccine and infection-generated antibody kinetics under a range of user specifications [14]. This may explain why field epidemiologists that perform serosurveys are often unaware of insights they can gain by applying these models to their datasets.

Here, we introduce *RSero*, an R package targeting this broad community that contains a comprehensive and user-friendly pipeline of methods to store datasets, analyze serological surveys, estimate FOIs, and compare different transmission models. The package implements widely used serocatalytic models as well as new models encompassing features such as multiple outbreaks. It also offers the possibility of incorporating seroreversion, to calibrate models to multiple independent datasets, and to account for different risks of infection when the surveys are collected in different populations. We also provide an illustration of the range of tools available for the broad community of field epidemiologists and modelers who may benefit from them.

## Materials and methods

The FOI can be estimated from age-stratified serological survey data using the classical theory of serocatalytic models [5]. We will always consider one year as the time unit. The probability that an individual of age *a* is seropositive depends on the FOI of the pathogen during their lifetime. For instance, a seropositive 1-year old child will have been exposed to the pathogen during the first year of their life. If we denote $\lambda_{1}$ the FOI during this first year of life, the probability that the child is seropositive at year 1 is $1-\text{exp}\left(-\lambda_{1}\right)$. More generally, the cumulative FOI of an individual of age *a* over their lifetime is related to the probability this individual is seropositive at sampling


$$P=1-\text{exp}(-\sum_{i=1}^{a}\lambda_{i}).$$ (1)

### Mode of transmission

We implemented several models describing different changes in the temporal dynamics of the FOI in the *Rsero* package. These include widely used models of constant or piecewise constant transmission, alongside new approaches to model outbreaks.

#### The constant model.

In the simplest model, the FOI is constant through time. Therefore, the probability that an individual of age *a* is seropositive is obtained by integrating the FOI over their lifetime and is equal to


P=1−exp(−a λ). (2)

#### The independent model.

In the independent model, the FOI is allowed to change every year. The probability of being seropositive at age *a* follows the general formula in eq (1) [6,15].

#### The piecewise-constant model.

The piecewise constant model extends the constant model to consider several periods of constant FOI. This model can be used for instance to describe the impact of an intervention which decreases the annual risk of infection in an endemic setting. For example, if the transmission was constant with annual FOI $\lambda_{A}$ before changing *T* years ago to an annual FOI $\lambda_{B}$, then the probability for an individual of age *a* to be seropositive is


$$P=1-\exp\left(-a\lambda_{B}\right)\text{ if }a\leq T$$

$$P=1-\exp\left(-a\lambda_{B}\right)\exp\left(-\left(a-T\right)\left(\lambda_{A}-\lambda_{B}\right)\right)\text{ if }a>T$$ (3)

#### The outbreak model.

We introduce an outbreak model that describes epidemic spread of a pathogen, i.e., assuming there have been *K* epidemics in the past, where *K* is fixed. The FOI at year *i* is given as a sum of *K* Gaussians. Each Gaussian is centered on *T*_*k*_, the peak epidemic time of the focal outbreak:


$$\lambda_{i}=\sum_{k=1}^{K}{\bar{\alpha }}_{k\;}exp\left(-{\left(i-T_{k}\right)}^{2}\right)\;\;$$ (4)

Where $\;{\bar{\alpha }}_{k\;}=\;\alpha_{k\;}\frac{1}{\sum_{i=0}^{A}exp\left(-{\left(i-T_{k}\right)}^{2}\right)\;}$.

In epidemic k, the FOI is maximal at the peak *T*_*k*_; $1-\text{exp}\left(-\alpha_{k\;}\right)$ is the attack rate (AR) of the outbreak, defined as the overall probability of infection over the course of the outbreak; In the case where the *T*_*k*_ are far apart, the Gaussians are well separated and around *T*_*k*_ the force of infection is given by a single Gaussian. When we assume a larger number of peaks than actually occurred, the overall yearly FOI is still well estimated and it is the sum of the individual ARs of the inferred epidemics that may overlap.

#### The outbreak + constant model.

In this model, the FOI is constant and punctuated by outbreaks. Similarly to the outbreak model, the FOI during year *i* is


$$\lambda_{i}=\lambda_{C\;}+\sum_{k=1}^{K}{\bar{\alpha }}_{k\;}exp\left(-{\left(i-T_{k}\right)}^{2}\right)\;$$ (5)

where $\lambda_{C\;}$ represents the constant part of the FOI.

### Seroreversion

Not all infections provide lifelong immunity. Models describing seroreversion (i.e., switching from seropositivity to seronegativity, or equivalently in this framework, from immune to susceptible) have been developed to study malaria [16] and other infectious diseases [17,18]. This phenomenon is captured by a seroreversion rate ρ. If the FOI is a constant λ, then the probability that an individual of age *a* is seropositive is given by


$$P\;=\;\;\frac{\lambda \;}{\lambda +\rho }\left(1-e^{-a\left(\lambda +\rho \right)}\right)$$ (6)

This formula has been extended for a FOI consisting of two constant phases [19]. Deriving the probability of seropositivity is not as straightforward for general models of time-varying FOI. In the *RSero* package, we introduce the possibility of combining seroreversion with any model of pathogen circulation.

The general formula for the probability for being seropositive for an individual of age *a* is not available in closed form but can be derived numerically. To do so, we reconstruct the yearly probabilities of infection or seroreversion during the time between birth and sampling. We note $X_{a}\left(t\right)\;$the probability that an individual born *a* years before the survey was seropositive *t* years before the survey. Starting at birth, where $X_{a}\left(a\right)\;=0$ (i.e., we assume all individuals are born seronegative), and considering the FOI on year *y* and the seroreversion rate *ρ*, the difference equation is


$$X_{a}\left(y-1\right)\;=\;X_{a}\left(y\right)\;e^{-\left(\lambda_{y\;}+\;\rho \right)}+\frac{\lambda_{y\;}}{\lambda_{y\;}+\;\rho }\left(1-e^{-\left(\lambda_{y\;}+\;\rho \right)}\right)$$ (7)

The probability that the individual is seropositive in the survey is thus $P=X_{a}\left(0\right)$.

### Imperfect sensitivity and specificity

The models can account for uncertainty in the seroprevalence estimates resulting from imperfect sensitivity and specificity of the assays. We note se and sp the sensitivity and specificity of the assay, respectively. In the scenario of perfect sensitivity and specificity (se=sp=1) the seroprevalence is the same as the proportion of the population infected by the pathogen. In the general case, if we denote this proportion $P_{inf}$, the probability for an individual to be observed as seropositive is


$$P=se*P_{inf}+\left(1-sp\right)*\left(1-P_{inf}\right)$$ (8)

In the implementation of the package, the user can give specific values to the sensitivity and specificity for any transmission models and with or without seroreversion.

### Setting different categories for the risk of infection

To account for differential susceptibility to a pathogen in a population, it is possible to estimate the FOI for different subgroups, defined by individual characteristics (e.g., sex, region).

If the serosurvey includes information about *n* categories, the probability for individual *j* to be seropositive is

$$P_{j\;}=\;1-exp\left(\;-\sum_{i=0}^{a_{j}}\lambda_{S-i}\times {f^{1}}_{{{\text{Cat}}^{1}}_{j}}\;\cdots \times {f^{n}}_{{{\text{Cat}}^{n}}_{j}}\right),\;$$ (9)

where $a_{j}$ is the age of the individual, which belongs to classes (${{Cat}_{j}}^{1},\ldots ,\;{{Cat}_{j}}^{n})$ in the categories 1...n. The FOI is multiplied by the parameters ${f^{k}}_{{{Cat}^{k}}_{j}}$ which describe the relative risk for ${{Cat}_{j}}^{k}$ compared to a defined reference group for which we assume ${f^{k}}_{{{Cat}^{k}}_{j}}$= 1.

### Serosurveys defined with a low age resolution

If individual ages are given in age groups, the likelihood is integrated over the possible ages. The probability for an individual whose age is between *a*_*1*_ and *a*_*2*_ to be seropositive is


$$\begin{array}{c} P_{j}=\frac{1}{L}\sum_{A=a1}^{a2}\left(1-exp\left(-\sum_{i=0}^{A}\lambda_{S-i}\right)\right)\\ \;=1-\frac{1}{L}\sum_{A=a1}^{a2}exp\left(-\sum_{i=0}^{A}\lambda_{S-i}\right), \end{array}$$ (10)

where L is the length in years of the age group [*a*_*1*_, *a*_*2*_] and is equal to *a*_*2*_*-a*_*1*_ *+1*.

### Parameter inference

The models are fitted to data using a Markov Chain Monte Carlo (MCMC) algorithm implemented in the *rstan* package [20], which provides an interface to Stan, a probabilistic programming language for specifying and fitting Bayesian statistical models. All results presented here were extracted from four independent chains of 5,000 iterations, with the first 2,500 corresponding to a warmup period. A No U-Turn sampler variant of Hamiltonian Monte Carlo was used to update the parameters. Convergence was assessed using functions implemented in the *rstan* package, including visual examination of the chains, Rhat statistics, the effective sample size (ESS), and the number of divergent transitions. In all examples provided here, we checked that the Rhat was less than 1.01, the number of divergences were low and the ESS was over 200 for all parameters. By default, the numerical sampling parameters were left at their default *rstan* values. We used a non-centered parameterization for the parameters when a normal or lognormal prior distribution was specified.

### Prior distributions

For each parameter, the user can choose and specify a Normal, Lognormal or an Exponential prior distribution. The default prior distributions and values of the hyperparameters used in the examples are as follows. For the outbreak models, and to constrain the parameters to be positive, the AR α_j_ are estimated with lognormal priors, defined as


$$\alpha_{j}=\alpha_{j}^{1}.\text{exp}\left(\alpha_{j}^{2}*\alpha_{j,raw}\;\right)$$

where $\alpha_{j,raw}\sim N\left(0,1\right)$ and by default, $\alpha_{j\;}^{1}=0.2$ and$\alpha_{j}^{2}=0.2.$

The peak times T_j_ are given by the non-centered parameterization


$$T_{j}=T_{j}^{1}+T_{j}^{2}*T_{j,raw}\;$$

where $T_{j,raw}\sim N\left(0,1\right)$ and by default, $T_{j\;}^{1}=20$ and$T_{j}^{2}=10.$

The FOI for both the constant and the independent model are $\lambda =\lambda^{1}.\text{exp}\left(\lambda^{2}*\lambda_{raw}\;\right)$, with the default prior parameters $\lambda^{1}=0.01$ and *λ*^2^ = 1. The prior parameters for the seroreversion rate are $\rho^{1}=1$ and $\rho^{2}=1$, with the parameterization $\rho =\rho^{1}.\text{exp}\left(\rho^{2}*\rho_{raw}\;\right)$. For the parameters characterizing the relative risks of infection for the different categories, a log normal prior distribution of mean 0 and variance 3 was chosen. This ensures that each category has the same risk of infection (e.g., the prior of the ratio group1/group2 is the same as the prior of the ratio group2/group1) [21]. Default priors are set to be weakly informative so they can represent any value of the seroprevaledance between 0 and 100% for all ages [22]. Prior predictive simulations are shown for various prior distributions for the outbreak model (S1 Fig) and the constant model with seroreversion (S2 Fig). We provided a guideline to help choose more informative priors based on the expected value of the seroprevalence, for various values of the annual FOI and the seroreversion rate (S3 Fig). Moreover, the impact of the prior distribution on the posterior distribution of the seroprevalence at various ages in shown in S4–S7 Figs.

Ninety-five percent credible intervals were calculated from the 2.5% and 97.5% percentiles of the posterior distributions.

### Model comparison

We implemented four model comparison methods that rely on a measure of out-of-sample prediction error [23,24]. These are the AIC, DIC, WAIC and PSIS-LOO, where the first two criteria are based on the deviance and the last two are based on computation of the log pointwise predictive density (LPPD). We recommend using the PSIS-LOO criterion, as it has become the preferred method in Stan [24]. This method evaluates the expected LPPD (ELPD) which is a Bayesian leave-one-out estimate of the LPPD and to which a standard error is associated. The loo_compare function in the loo package compares the ELPD and it associated SE for the different models and was used for model selection.

### Description of the package

The *Rsero* package is written in R. The code is available on GitHub https://github.com/nathoze/Rsero along with information on how to install the package, tutorials and descriptions of the functions. We describe here the four main components of the package: the data, the model, the fitting procedure, the posterior analysis.

#### The data.

One of the features of *Rsero* is an ability to handle and store serological data in a standard format called SeroData to allow for further analysis and fit with serocatalytic models. A serological survey contains primarily the age and seropositivity status of the individuals, and can also contain information such as the year of sampling and categories such as sex and location that are related to different risks of infection. Users can combine the serosurveys conducted at different time points or those with different groupings of age.

#### The model.

A second important feature is the choice of a model to reconstruct the historical force of infection from the serological data. The FOImodel function sets up the different components of a model of the yearly variation of the FOI. This function takes a first argument type that accounts for the dynamics of the FOI. For instance, if the FOI is constant, the model is defined as:

model = FOImodel(type=‘constant’)

The other possible models include independent, outbreak, piecewise constant (piecewise), combination of constant FOI and outbreaks (constantoutbreak). In those models the number of peaks and constant phases are to be specified by the user. Furthermore, it is in the FOImodel class that the user can specify additional information regarding the serology: accounting for seroreversion is done with an entry seroreversion = TRUE in the FOImodel definition; the sensitivity and specificity of the assays are set to fixed values specified by the user, with default values se = 1 and sp = 1. The user can also specify the parameters of the priors.

#### The fit.

Inference of the serocatalytic models is done with a Bayesian approach using MCMC methods. We implemented in *RSero* a fit function that requires as parameters at least the serological data and the serocatalytic models to estimate the FOI (and other parameters if necessary). More generally, fit is written as a wrapper for the *rstan* function sampling and as such can be given the parameters of this function, such as the number of iterations, the number of chains, the initial parameters, the sampling algorithm, etc. In the simplest case, it is called as:

model.fit = fit(data = data, model = model)

#### The posterior analysis.

Finally, once sampling has been done the user can visualize the fit of the FOI, the traceplots for each parameter, the posterior distribution, estimate the convergence using tools implemented in *rstan*, and compute the different information criteria.

## Results

We showcase here how to use the *Rsero* package to estimate parameters of serocatalytic models on simulated serological surveys and real-life examples. Each example is accompanied with the R code for data management, model definition, inference and post hoc analysis to follow the step-by-step usage of the package.

### Characterizing the appropriate model of circulation

#### Model comparison in a simulation study.

**Fig 1:**
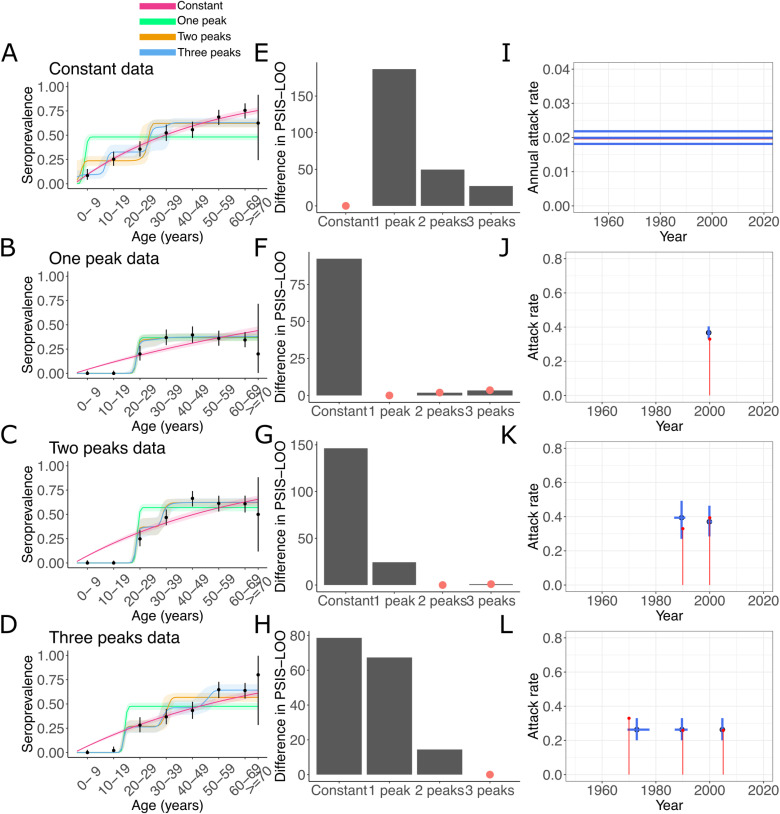
(A–D) Simulations of the proportion of seropositive individuals, by age group, and reconstruction with a model of constant transmission and of 1, 2 and 3 outbreaks. The line and envelope represent the mean and 95% credible interval of the posterior distribution. (E–H) Identification of the model of circulation by comparing the PSIS-LOO of the models. A difference larger than 5 indicates a weak support for the model. Models with a difference lower than 5 compared to the best model are indicated with a red dot. (I) Posterior estimates of the annual AR for the constant model. (J–L) Posterior estimates of the peak year and the total ARs during the outbreaks for the 1 to 3-outbreak models (blue). Mean and 95% credible intervals are represented. Red: input value used to simulate the data.

To test the ability of our approach to select for the correct epidemiological scenario, we simulated serosurveys for each of the following scenarios: one constant annual force of infection and three outbreaks with one to three successive peaks. A total of 1000 individuals with age 1 to 70 yo were included in each survey. We fitted each of the four serocatalytic models to the data (Fig 1A–D). Visually and based on PSIS-LOO, we were able to identify the correct model of transmission for each dataset (Fig 1E–H). However it was not always possible to exclude other circulation models. For instance, the seroprevalence in the scenario of two successive peaks was also well explained by the model with three peaks (Fig 1C and G).

We estimated the model parameters using the model with the best score (and the most parsimonious among two equally good models). The AR and the epidemic years were well estimated (Fig 1I–L).

We now show and comment the R script to run the above example. We first generate the four surveys corresponding to the different circulation scenarios. This is done with a *Rsero* function called simulate_SeroData which creates a SeroData object.

data_peak1 = simulate_SeroData(foi = 0.4, 

             sampling_year = 2023,

             epidemic_years = 2000,

             number_samples = 1000)

data_peak2 = simulate_SeroData(foi = c(0.4,0.5), 

             sampling_year = 2023,

             epidemic_years = c(1990,2000),

             number_samples = 1000)

data_peak3 = simulate_SeroData(foi = c(0.4,0.3,0.3), 

             sampling_year = 2023,

             epidemic_years = c(1970,1990,2005),

             number_samples = 1000)

data_constant = simulate_SeroData(foi = rep(0.02, 70) ,

             sampling_year = 2023,

             epidemic_years = seq(1954, 2023),

             number_samples = 1000)

We then define the different circulation models we want to apply to the data with the *Rsero* function FOImodel that takes the type of model (constant, outbreak, combination of constant and outbreak, etc.) as an input as well as other parameters, such as the number of peaks for the outbreaks model.

model_peak1 = FOImodel(type = 'outbreak', K=1)

model_peak2 = FOImodel(type = 'outbreak', K=2)

model_peak3 = FOImodel(type = 'outbreak', K=3)

model_constant = FOImodel(type = 'constant')

We then fit the different models to each dataset. For instance, here we fit the model with two outbreaks to the data that corresponds to the constant FOI using the *Rsero* function fit, compute the PSIS-LOO and plot the fitted seroprevalence along with the data.

fit_model= fit(data = data_constant,

             model = model_peak2,

             chains = 4,

             cores = 4, 

             iter = 20000)

C = compute_information_criteria(fit_model)

print(C$PSIS_LOO)

seroprevalence.fit(fit_model)

#### Application to a serosurvey of Chikungunya in the Philippines.

**Fig 2:**
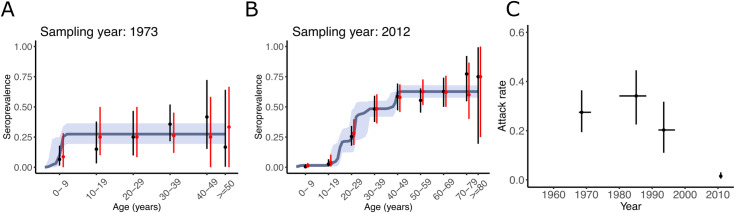
Reconstruction of Chikungunya outbreaks in the Philippines. (A and B) Mean and 95% binomial confidence interval of the seroprevalence (black), posterior distribution of the seroprevalence and posterior predictive distribution (red) for the 1973 and 2012 surveys. (C) Posterior distribution of the AR and epidemic years in the period 1955-2012 in a model of four outbreaks. The intervals are the 95% credible intervals of the AR and epidemic years.

We apply this approach to real-world data from the Philippines, a country that experienced successive Chikungunya outbreaks over at least the last 60 years. This dataset is a combination of two cross-sectional studies both obtained in Cebu city. One study was conducted in 1973 and included 150 individuals, including children and adults older than 60 y.o. Data was aggregated in 10-year age groups. The second study was conducted in 2012 and included 853 participants randomly sampled in the population including children >6 months of age. These studies were previously analyzed with serocatalytic models in [6], using an approach relatively similar to the independent model described above with outbreak years being defined as years with an annual FOI >1%. Here, we use our outbreak model to obtain a more parsimonious description of the underlying epidemic process. We evaluated the number of peaks using the PSIS-LOO and in agreement with [6], we identified four successive outbreaks (Fig 2A and B). We found an outbreak contemporary to the most recent survey with an AR of 1.5% (95% CrI: 0.5%–3.1%) (Fig 2C), as well as outbreaks in 1993 (95% CrI: 1992-1997) (AR= 20%; 95% CrI: 11%–32%), in 1985 (95% CrI: 1980–1988) (AR=34%; 95% CrI: 23%–45%) and 1969 (95% CrI: 1968–1971) (AR=27%; 95% CrI: 19%–36%).

### Accounting for risk factors

**Fig 3:**
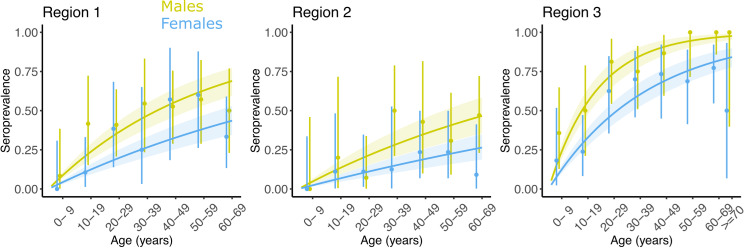
Model fit to simulated seroprevalence data by age group, sex and region for a constant model of transmission.

We show how the package can be used to estimate differences in the FOI between groups. We simulated a seroprevalence survey where the 600 participants were categorized according to sex and location (male/female and Regions 1, 2 and 3), assuming constant transmission. We were able to accurately reproduce the seroprevalence data in all regions (Fig 3). Moreover, taking males and Region 1 as the reference in their respective category, we retrieved the relative risks of infection. FOI of females was found to be 0.60 (95% CrI: 0.45–0.77) that of males (input = 0.50), whereas we found the FOI in Region 2 relative to Region 1 to be 0.58 (95% CrI: 0.38–0.82) (input = 0.50) and in Region 3 relative to Region 1 to be 3.79 (95% CrI: 2.77–5.14) (input = 3.0).

The R script to run the above example is given below. We first simulate a serosurvey where we specify the sex and location of the participants

foi = 0.018

n_samples = 600 

sex.list = c('males', 'females')

location.list = c('Region 1', 'Region 2', "Region 3")

sex <- sample(sex.list, n_samples, replace = TRUE)

loc <- sample(location.list, n_samples, replace = TRUE)

risk_factor = 1*(sex=='males') + 0.5*(sex=='females') 

risk_factor = risk_factor*(1*(loc=="Region 1")+

                    0.5*(loc=="Region 2")+

                    3*(loc=="Region 3"))

ages = round(runif(n=length(sex))*70)+1

data = SeroData(age_at_sampling = ages,

             Y = runif(n = n_samples) < 1-exp(-foi*risk_factor*ages), 

             sampling_year = 2023,

             sex = sex,

             location = loc,

             category = cbind(sex, loc))

Then we define the model we want to test (a constant FOI model here) and fit it to the data. The fitted parameters are then accessible using the *rstan* function extract, which extracts samples from the fitted model.

model = FOImodel(type='constant')

fit = fit(data = data,

     model = model,

     chains = 4, cores = 4, iter = 20000)

Chains = rstan::extract(fit$fit)

### Determining the impact of an intervention

**Fig 4:**
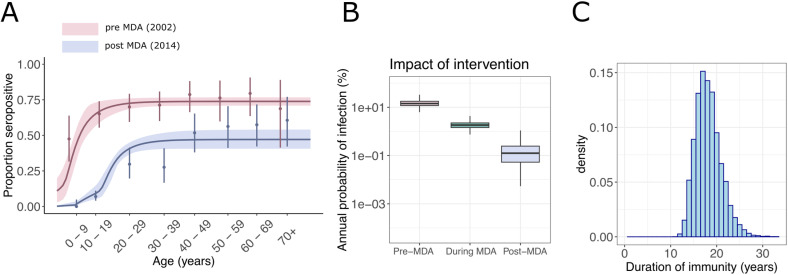
Impact of mass-drug administration on Trachoma prevalence in Nepal. (A) C. Trachomatis seroprevalence in Nepal pre- and post-MDA (respectively in red and blue), for CT694 antigen. The serocatalytic model used here assumed the succession of three constant phases of infection and seroreversion. (B) Annual probability of infection before, during and after the intervention. (C) Posterior distribution of the duration of immunity.

To evaluate the impact of mass drug administration (MDA) on Trachoma transmission in Nepal, serological surveys were conducted before (2002) and after (2014) the campaign that started in 2007[17,25]. 473 and 619 participants aged 1-90 y.o. were recruited, respectively. The serological surveys show a clear decrease in seroprevalence by age following the intervention in the younger age group (Fig 4A), but also a lower plateau in older participants.

Different models were fitted to pre- and post-MDA serosurveys simultaneously. Using the PSIS-LOO criterion, we showed that the model with three consecutive piecewise-constant FOI and seroreversion provided a better fit to the data than the same model without seroreversion and the model with two piecewise-constant FOI and seroreversion. This suggests endemic circulation in the past followed by a progressive decrease of the FOI before the implementation of the MDA, followed by a period of much lower transmission after the MDA. We estimate that the annual probability of infection dropped from 16.3% (95% CrI: 8.4%–40.0%) before 1998 (95% CrI: 1994–2000) to 1.9% (95% CrI: 0.79%–3.3%) between this time and 2007, and 0.17% (95% CrI: 0.005%–0.58%) after the intervention (Fig 4B). The duration of immunity, measured here as the half-life of seropositivity, was estimated at 18.2 years (95% CrI: 13.7 years–24.5 years) (Fig 4C). The discrepancy between the model and the data suggests that there might be additional explanation for the important decrease in the seroprevalence, including socioeconomic improvement in the community [25].

If sero_pre and sero_post are the serological surveys before and after the MDA, respectively, we combine them in a new SeroData object. In this object the sampling year of each participant is known. Then, we define a piecewise model with parameter *K* = 3, which is a piecewise constant FOI with three phases, and then fit the model.

data = combine_surveys(sero_pre, sero_post)

model = FOImodel(type = "piecewise", K=3, seroreversion = TRUE)

fit = fit(data = data, model = model)

### Applying the framework to optimize the design of serological studies

We illustrate here how the package can be used to optimize study design under various epidemiological scenarios. In particular, we determine the respective advantages of recruiting in the general population or only among children. Assuming a constant FOI, we conducted a comparative analysis of FOI estimates for different study sizes - from 100 to 1000 individuals - and for different epidemiological scenarios with FOI ranging from 0.01 to 0.2 (approximately 1% to 20% chance of being infected a given year). We considered studies with three different target populations: 1) individuals under 10 years old, 2) individuals under 20 years old, and 3) the general population (up to 70 years old).

**Fig 5:**
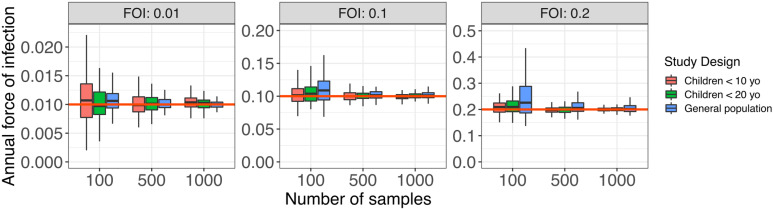
Impact of sampling strategy on FOI estimation. A serocatalytic model with constant transmission was used to estimate the posterior mean of the FOI over 100 simulated surveys for each set of parameters. The red line is the input FOI.

For each scenario, we simulated 100 studies. The input FOI value always fell in the 95% confidence interval (Fig 5). Larger sample sizes provided narrower confidence intervals. The study design minimizing uncertainty (i.e., narrower credible intervals) depends on the expected FOI. It is better to sample in the general population for low FOI and in children for high FOI.

**Fig 6:**
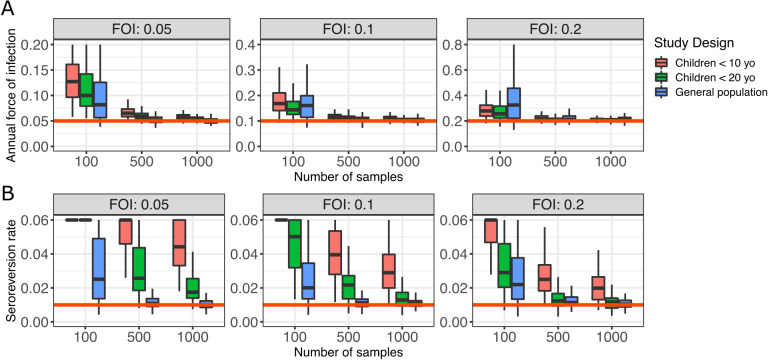
Impact of sampling strategy on the estimation of the seroreversion rate and the FOI. A serocatalytic model with constant transmission and seroreversion was used to estimate the posterior mean of the FOI (A) and the seroreversion rate (B) over 100 simulated surveys for each set of parameters. The parameter *ρ* was set to 0.01. The red line represents the input FOI (A) and seroreversion rate (B).

We then extended the analysis for models of constant FOI with seroreversion. For all age structures of the sampled population both the FOI and the seroreversion rate were overestimated when the number of samples was small (Fig 6). Adding samples improved the FOI estimate. However, it was not enough to provide a good estimate of the seroreversion rate in all surveys because this required that the study design included samples from all age groups. Indeed, data from younger individuals is informative for the rate of infection, whereas older individuals that have experienced infections and waning of their immunity are essential to estimate the seroreversion rate (Fig 6B).

## Discussion

In this paper, we showcase how serocatalytic models can be used to gain insight from age-specific seroprevalence data. We introduce Rsero, a new user-friendly R package, so that these methods become readily available to a broad audience of epidemiologists and modellers. Such open-source tools are important to ensure new methodological developments lead to effective improvements in the analysis of age-stratified serological data.

A large choice of models to characterize the historical variations of the FOI are implemented in the package. The independent model is the less parcimonious one as it provides one estimate the FOI per year. We propose more parsimonious models, such as the outbreak model, that has the advantage to also offer a direct interpretation of the timing of infection and the number of outbreaks that a population experienced. Although we cover a number of epidemiological scenarios within the FOIModel class, advanced users may also develop their own *rstan* functions to propose other models of circulation. Additionally, including seroreversion and imperfect serological assays to new circulation models is straightforward.

Model comparison is facilitated by this unified framework of circulation models and the *compute_information_criteria* function can be used with minimal changes if a new model was to be implemented. We implemented the AIC, DIC, WAIC and PSIS-LOO as these criteria are commonly used in the community but users may adapt the *compute_information_criteria* function to implement new criteria and test their models. We chose and recommended the PSIS-LOO as the baseline comparison metrics as it is fully Bayesian, integrating over the posterior distribution. This contrasts with the commonly used DIC which uses the mean of the deviance. Moreover, DIC is not suitable if the posterior is not Gaussian.

Users have the option to choose between normal, uniform, and exponential priors, with normal priors set as the default. These priors are specified within the Stan function, and users cannot directly specify other prior distributions from the R interface. However, the current functions are flexible enough to define both strongly informative and weakly informative priors. Generally, we recommend using the default normal priors as they help stabilize computations. In contrast, uniform priors often lead to divergences, even when other convergence criteria such as Rhat and ESS indicate excellent convergence.

Serocatalytic models work particularly well for diseases that provide long-lasting and protective antibodies, as they can estimate the FOI for outbreaks that occurred years before the survey. We showed in a simulation study (Fig 1) that parameters of past outbreak were well recovered even though few participants of the survey were already born when the outbreak occurred. This is also the case for Chikungunya, that we use as an example here. In the case of infections that do not generate sustainable antibody responses, multiple cross-sectional surveys conducted at well-separated intervals can improve the inference, but we also demonstrate that a well-designed single cross-sectional survey can be effective even when seroreversion is significant.

One advantage of the package is that it simplifies the management of serological surveys to easily join datasets and explore the impact of risk factors. Moreover, it is easy to simulate synthetic serosurveys acquired in various user-defined epidemiological settings. We show how these simulations allowed us to use the package to choose the number of samples and the age of the studied population depending on the specified scenario.

We focused here on binary status of seroprevalence principally because they are common in past and currently acquired data. However, in recent years, quantitative antibody data have led to tremendous advances in the understanding not only of the history of infections, but also in a more accurate definition of epidemic risk through the study of individual antibody dynamics [26], in the joint reconstruction of infection history and immune responses to an infection [7,13,27].

We believe that the publication of Rsero will facilitate and improve the analysis of serological data with serocatalytic model by all, benefiting both modellers and epidemiologists interested in such data.

## Supporting information

S1 Fig Prior predictive simulations of the age-profile of seroprevalence to assess the appropriateness of the prior distributions for the parameters a and T used in the outbreak model. The shaded area is the 95% highest density interval and the solid line is the mean estimate of seroprevalence. The priors were specified as follows: (A) *α* is Lognormal(log(0.2), 1) and T is Normal(30, 30). (B) *α* is Lognormal(log(0.4),1) and T is Exponential(1/20). (C) *α* is Lognormal(log(0.2),1) and T is Normal(10, 4). (D) *α* is Exponential(10) and T is Normal(30, 30).
(PDF)

S2 Fig Prior predictive simulations of the age-profile of seroprevalence to assess the appropriateness of the prior distributions for the parameters l and r used in the constant model with seroreversion. The shaded area is the 95% highest density interval and the solid line is the mean estimate of seroprevalence. The priors were specified as follows: (A) *λ* is Lognormal(log(0.01), 1) and *ρ* is Lognormal(log(0.01), 1). (B) *λ* is Exponential(10) and *ρ* is Lognormal(0, 1). (C) *λ* is Lognormal(log(0.01), 1) and *ρ* is Exponential(10). (D) *λ* is Exponential(1) and *ρ* is Exponential(1).
(PDF)

S3 Fig Age-profile of seroprevalence for various values of the parameters *λ* and *ρ* in the constant model and in the constant model with seroreversion. (PDF)

S4 FigPrior distribution of lambda (blue, normal distribution with parameters $\lambda^{1}=0.01$ and $\lambda^{2}=0.2$ ) and posterior distribution of the seroprevalence at age 20, 40 and 60 y.o. (red).(PDF)

S5 FigPrior distribution of lambda (blue, normal distribution with parameters $\lambda^{1}=0.01$ and $\lambda^{2}=1$ ) and posterior distribution of the seroprevalence at age 20, 40 and 60 y.o. (red).(PDF)

S6 FigPrior distribution of lambda (blue, normal distribution with parameters $\lambda^{1}=0.05$ and $\lambda^{2}=0.2$ ) and posterior distribution of the seroprevalence at age 20, 40 and 60 y.o. (red).(PDF)

S7 FigPrior distribution of lambda (blue, normal distribution with parameters $\lambda^{1}=0.05$ and $\lambda^{2}=1$ ) and posterior distribution of the seroprevalence at age 20, 40 and 60 y.o. (red).(PDF)
