# Supplementary figures and images for: RSero: A user-friendly R package to reconstruct pathogen circulation history from seroprevalence studies
Source: PLoS Comput Biol. 2025 Feb 3;21(2):e1012777. doi: 10.1371/journal.pcbi.1012777 (PMC11809794; doi:10.1371/journal.pcbi.1012777)

**A**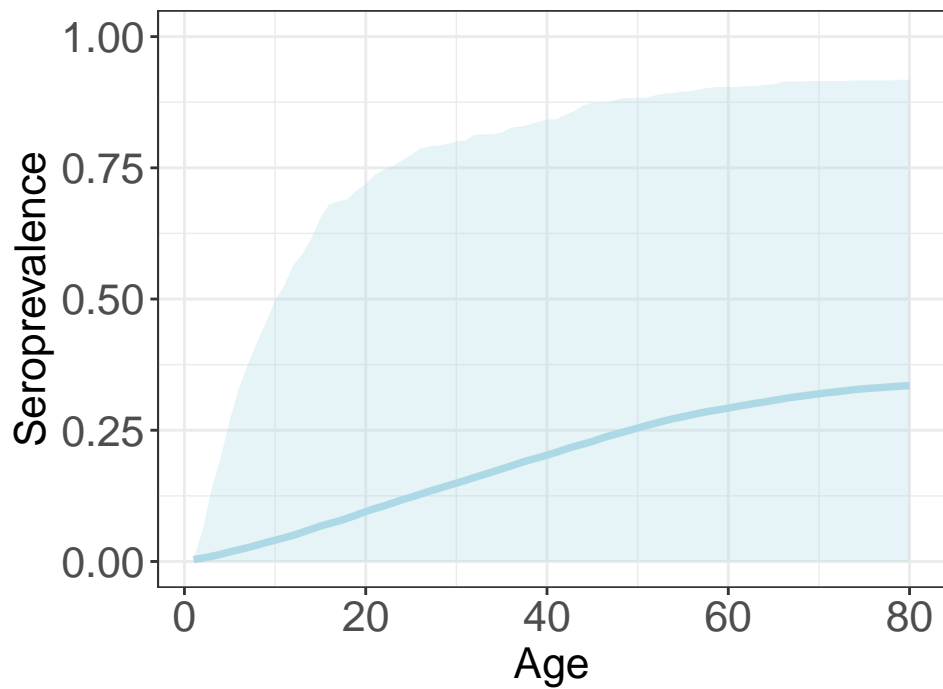**B**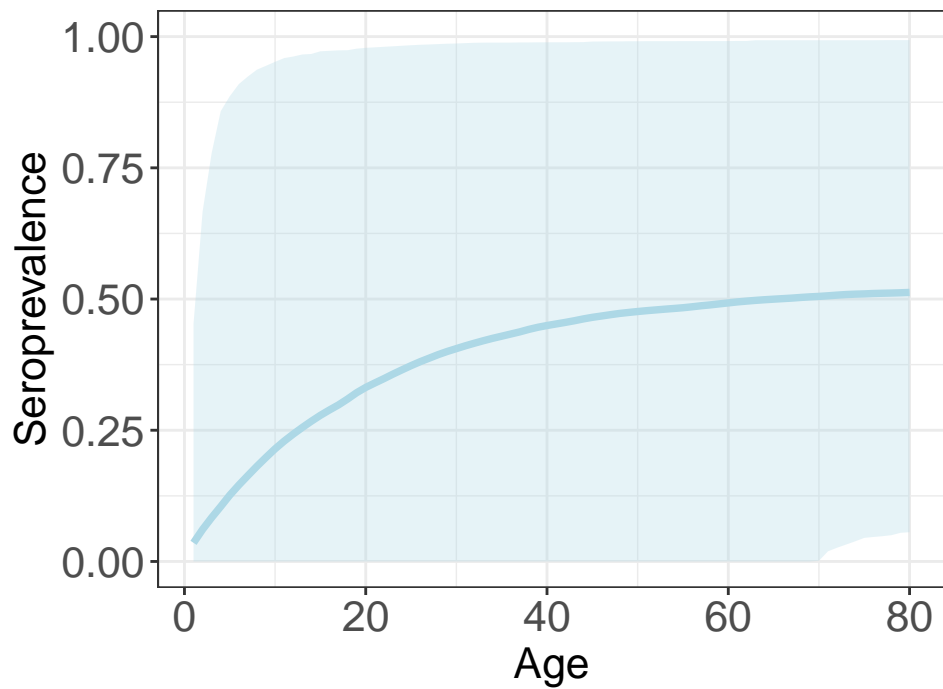**C**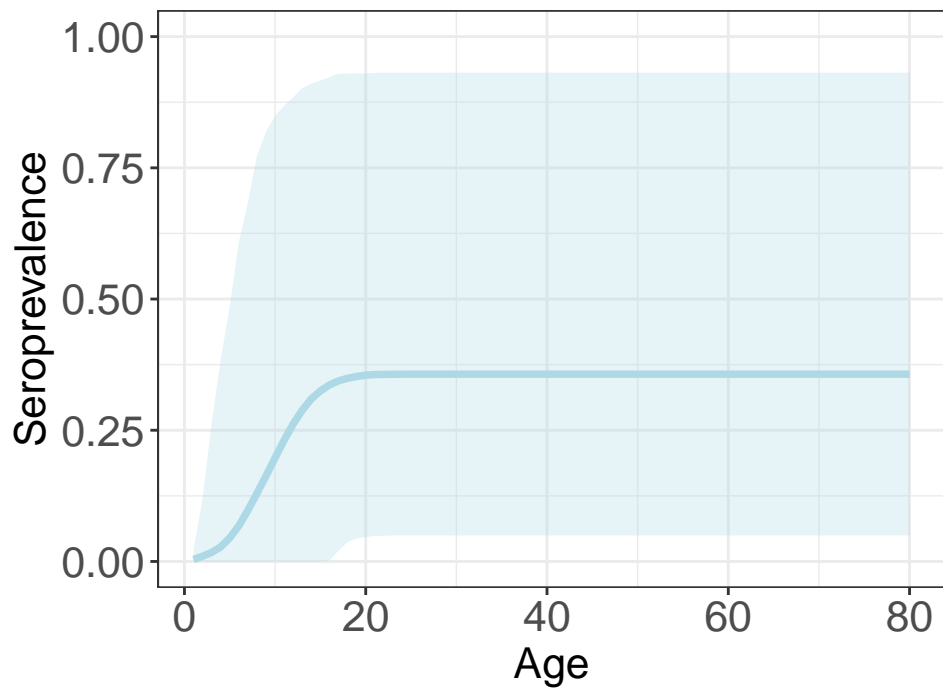**D**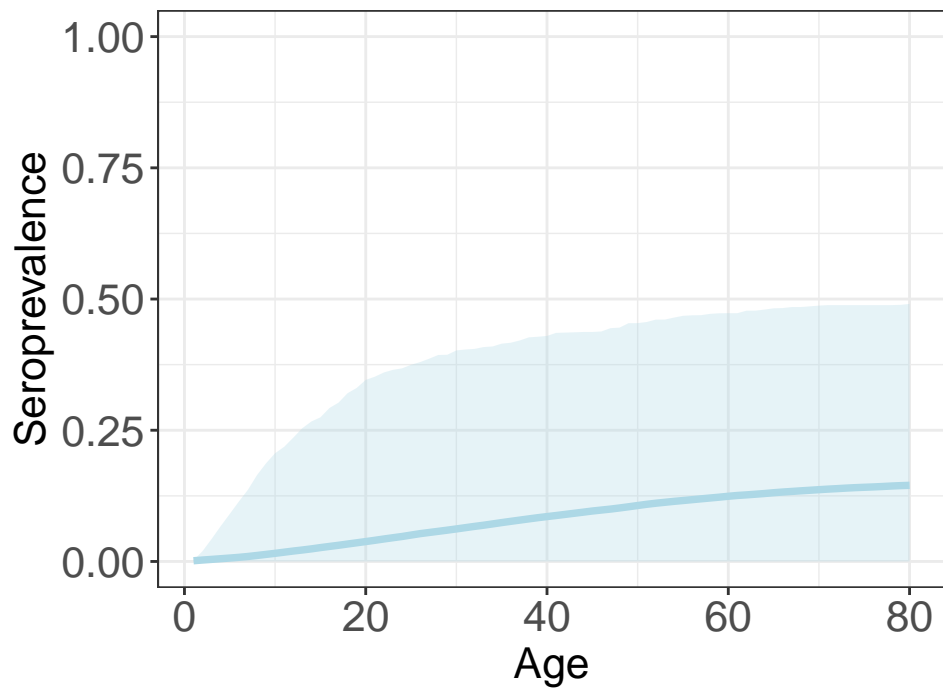

Supplement: S1 Fig — The shaded area is the 95% highest density interval and the solid line is the mean estimate of seroprevalence. The priors were specified as follows: (A) α is Lognormal(log(0.2), 1) and T is Normal(30, 30). (B) α is Lognormal(log(0.4),1) and T is Exponential(1/20). (C) α is Lognormal(log(0.2),1) and T is Normal(10, 4). (D) α is Exponential(10) and T is Normal(30, 30). (PDF) [file pcbi.1012777.s001.pdf]

**A**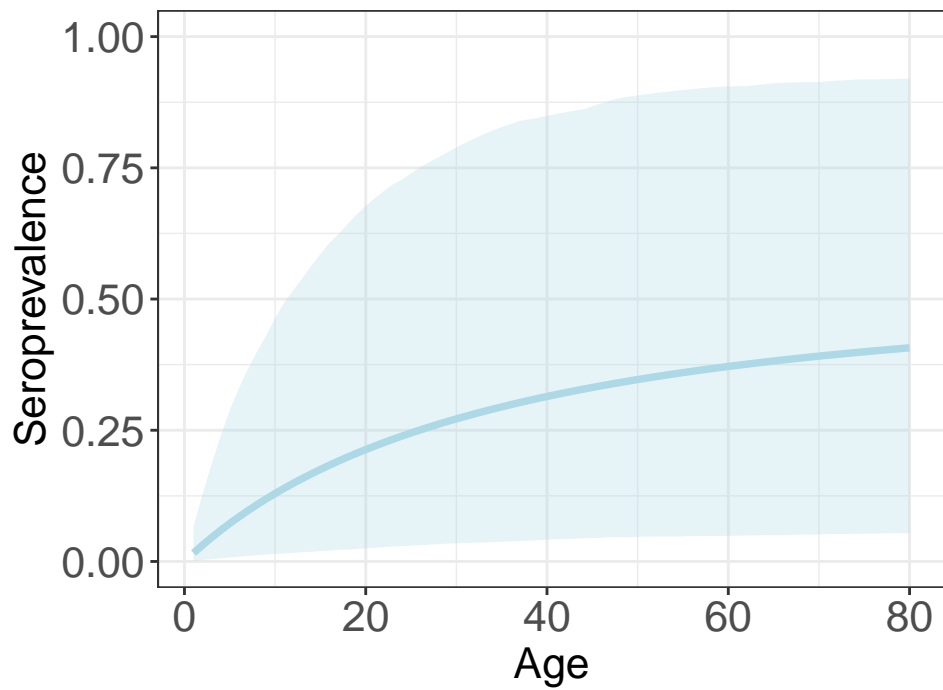**B**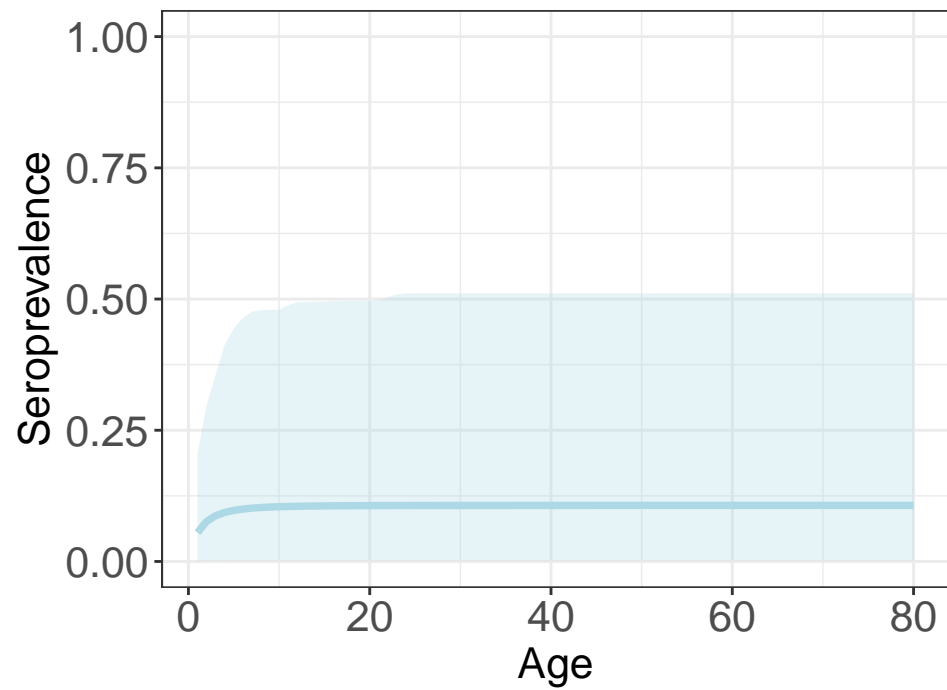**C**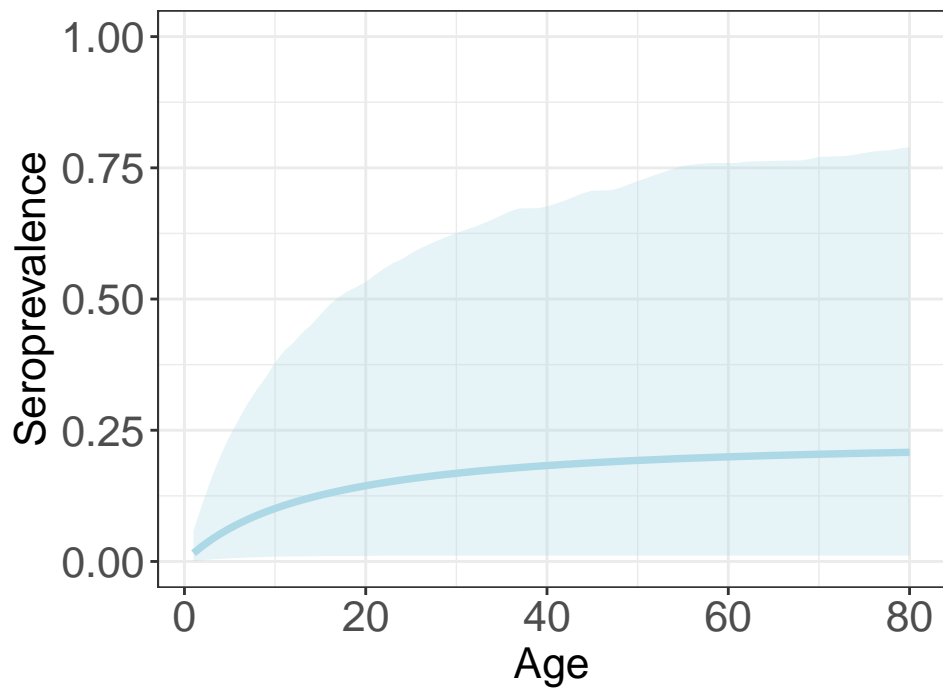**D**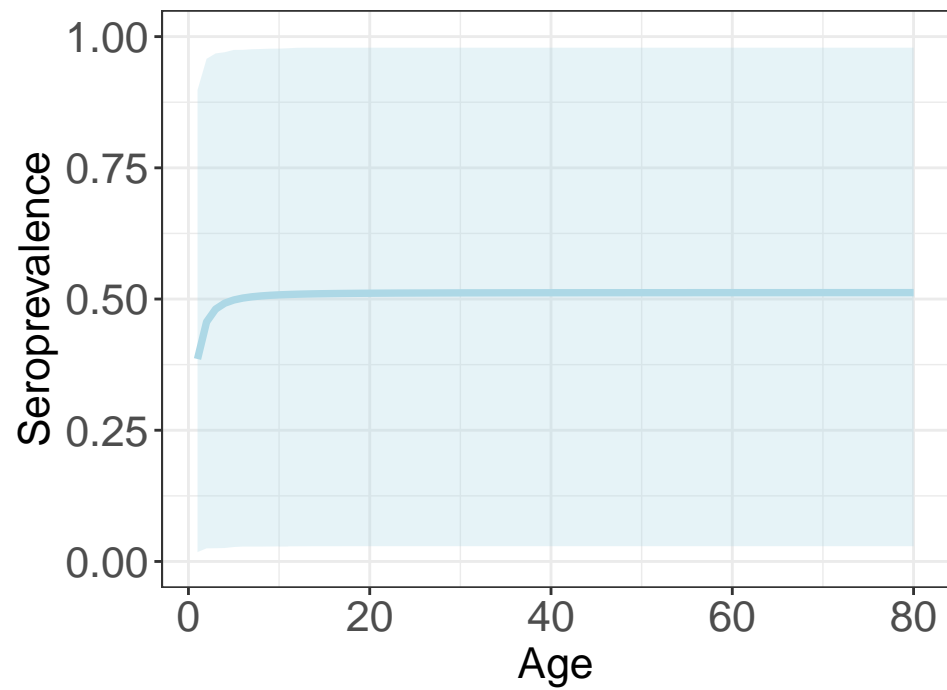

Supplement: S2 Fig — The shaded area is the 95% highest density interval and the solid line is the mean estimate of seroprevalence. The priors were specified as follows: (A) λ is Lognormal(log(0.01), 1) and ρ is Lognormal(log(0.01), 1). (B) λ is Exponential(10) and ρ is Lognormal(0, 1). (C) λ is Lognormal(log(0.01), 1) and ρ is Exponential(10). (D) λ is Exponential(1) and ρ is Exponential(1). (PDF) [file pcbi.1012777.s002.pdf]

Constant

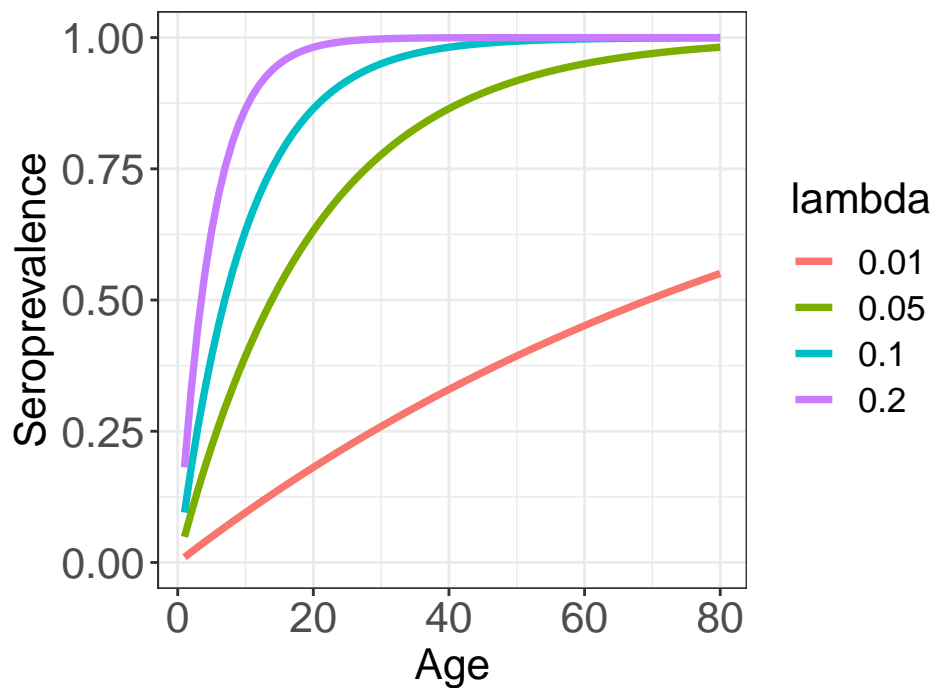Seroreversion,  $\rho = 0.05$ 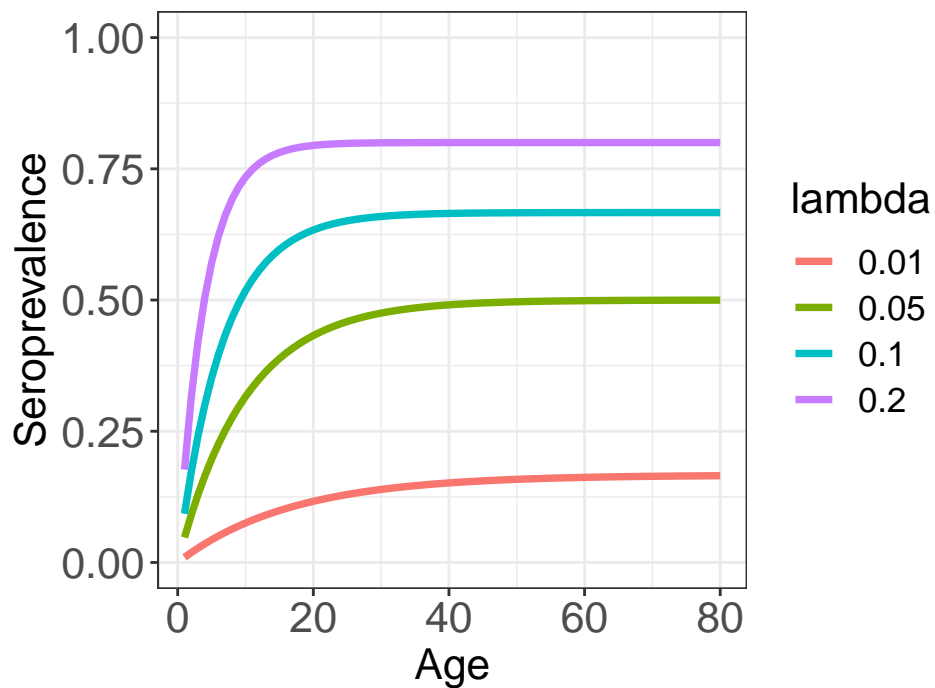Seroreversion,  $\rho = 0.1$ 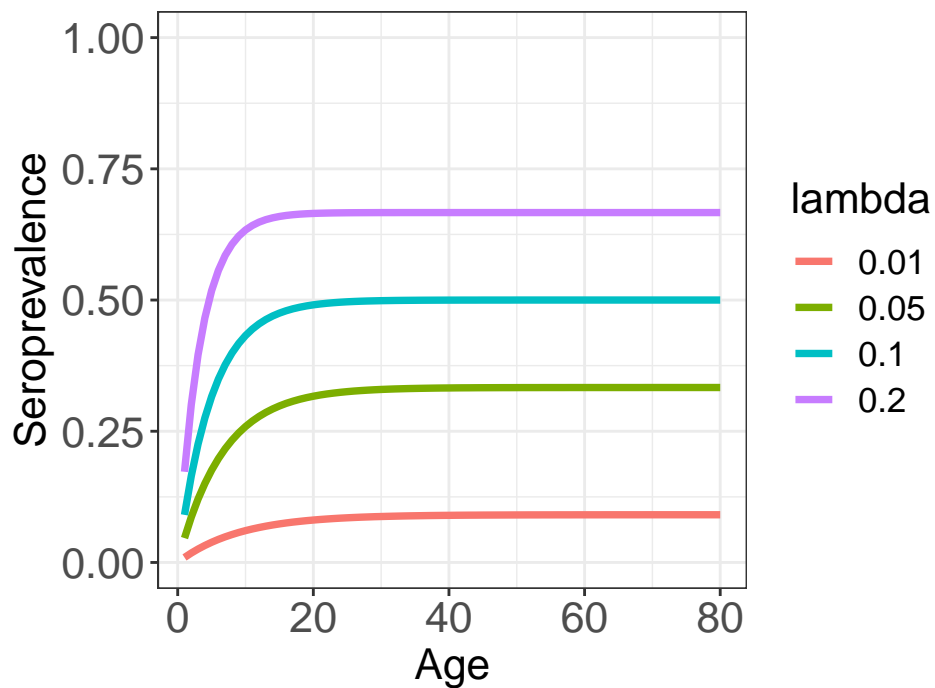Seroreversion,  $\rho = 0.2$ 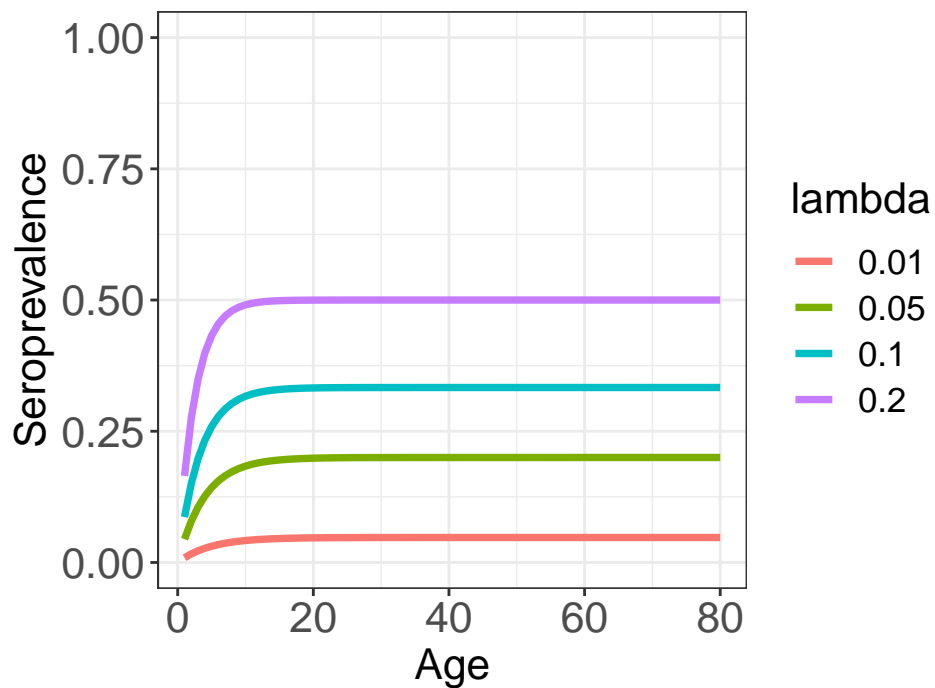

Supplement: S3 Fig — (PDF) [file pcbi.1012777.s003.pdf]

lambda1: 0.01, lambda2: 0.2

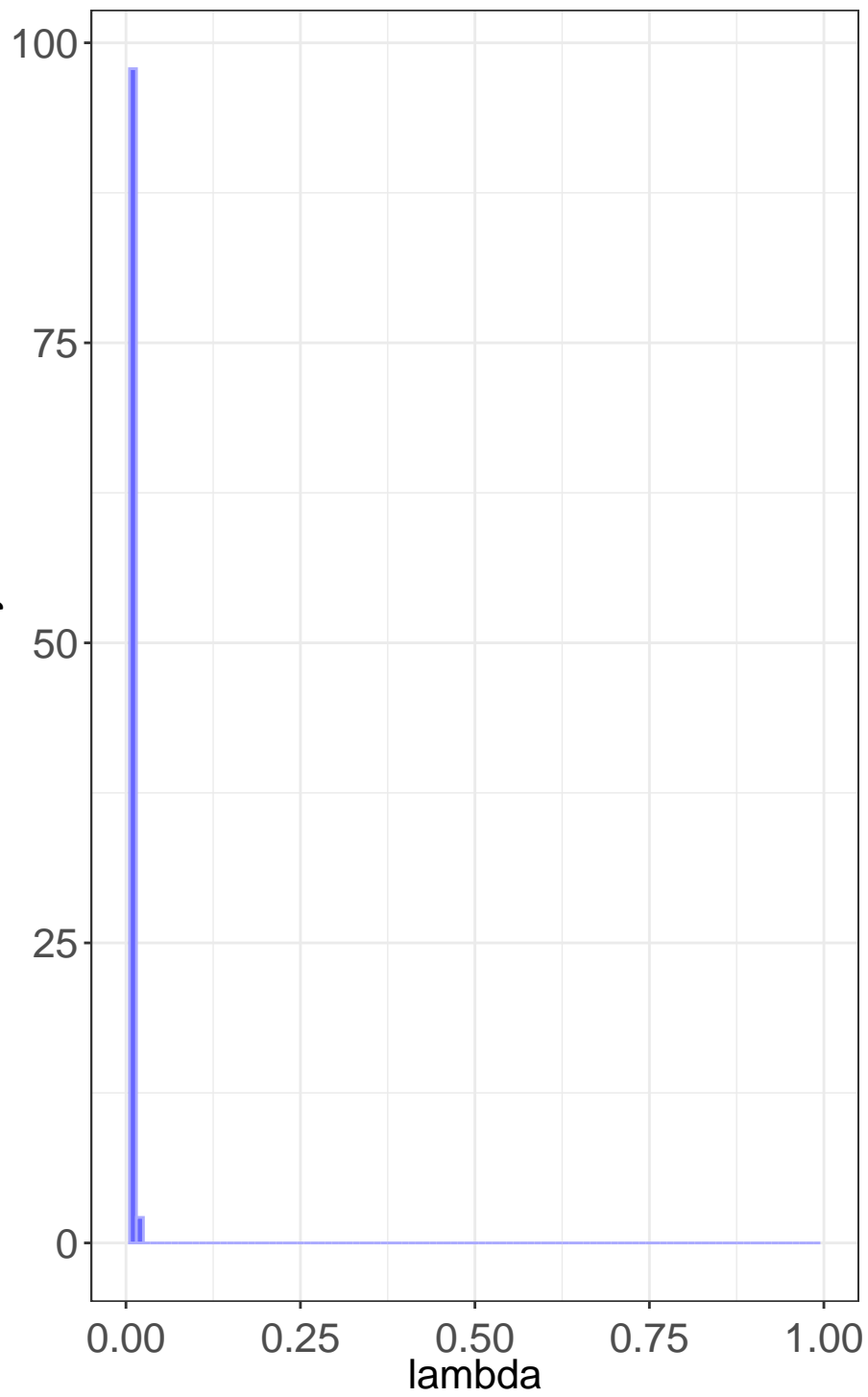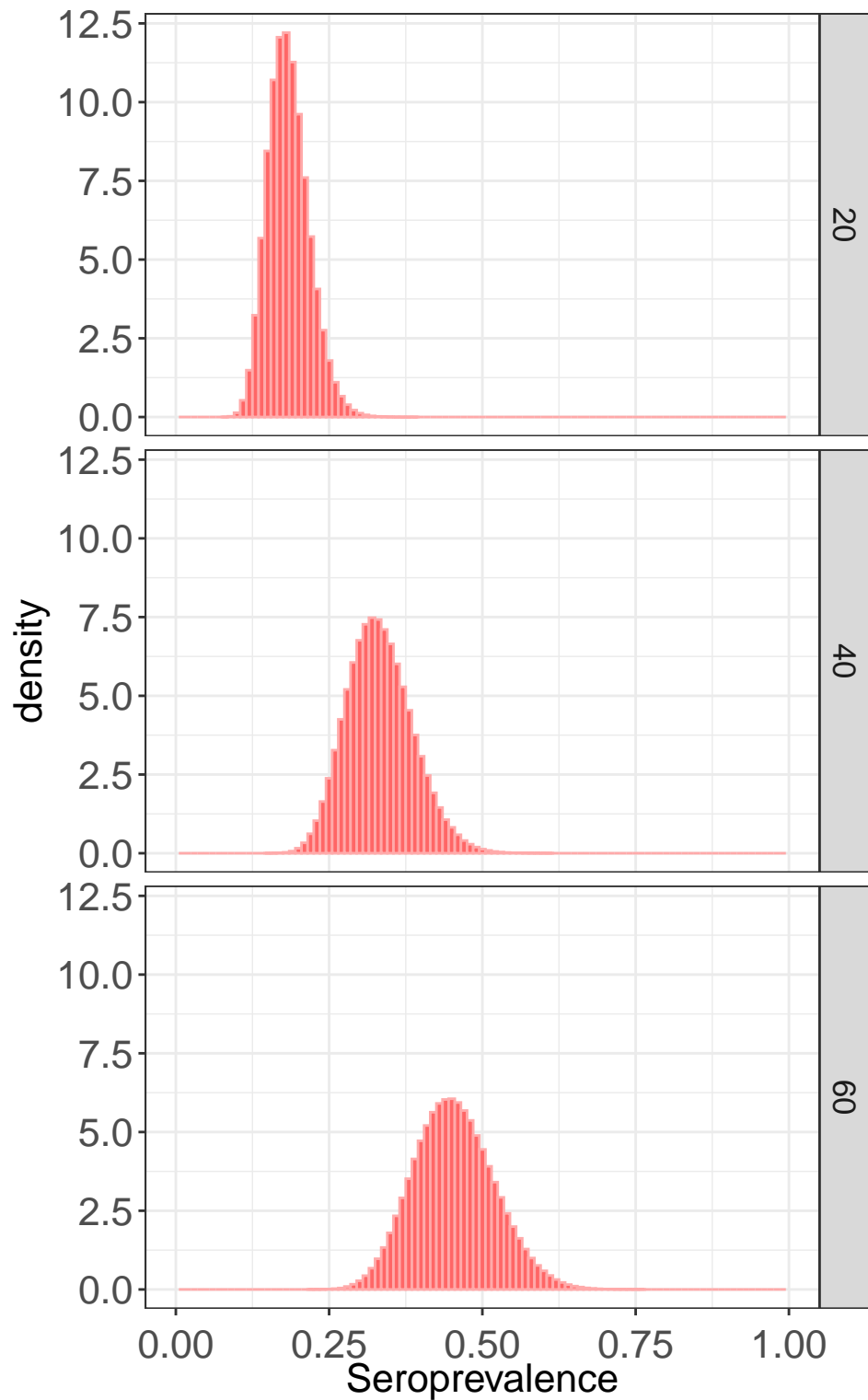

Supplement: S4 Fig — (PDF) [file pcbi.1012777.s004.pdf]

lambda1: 0.01, lambda2: 1

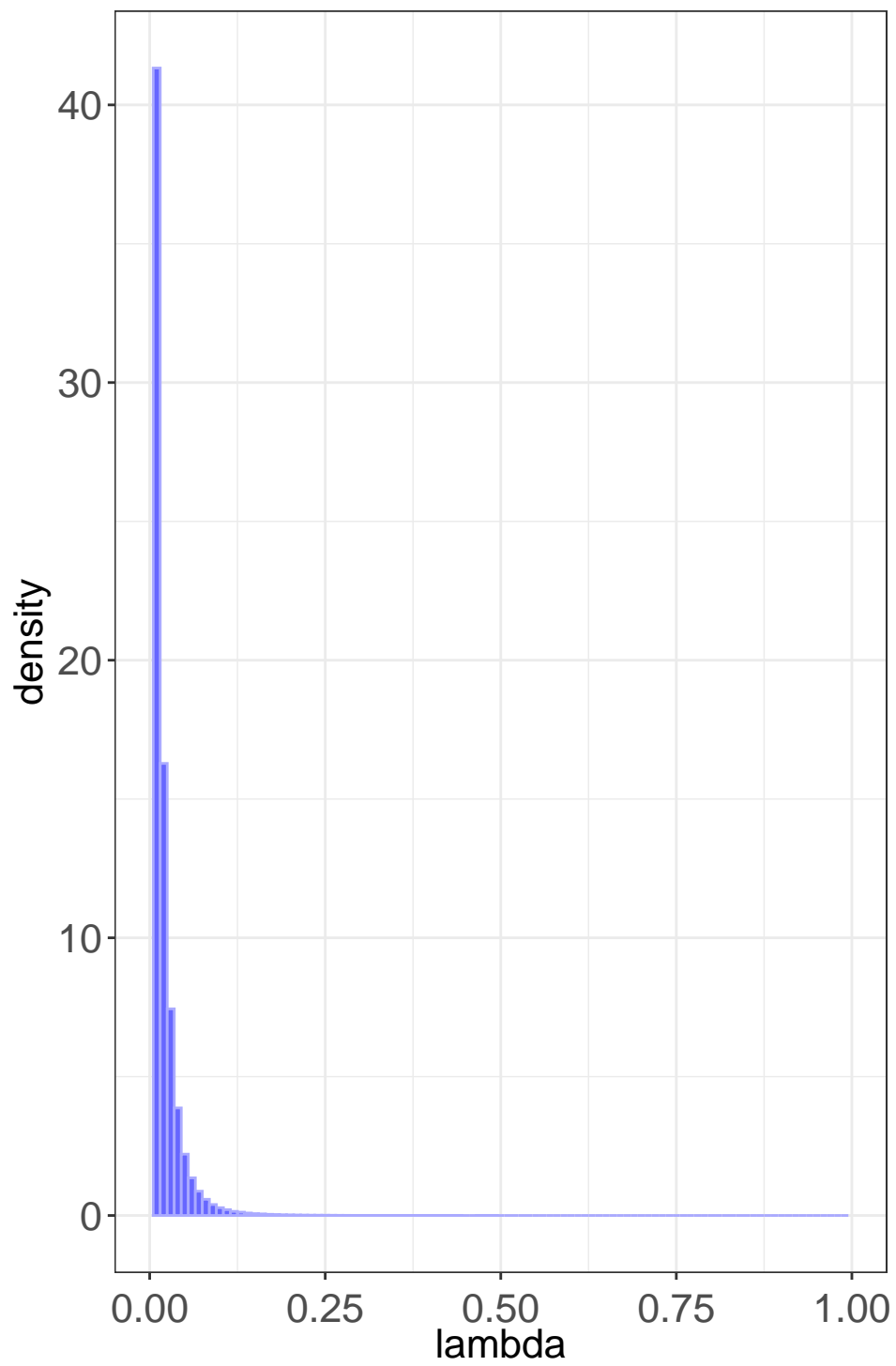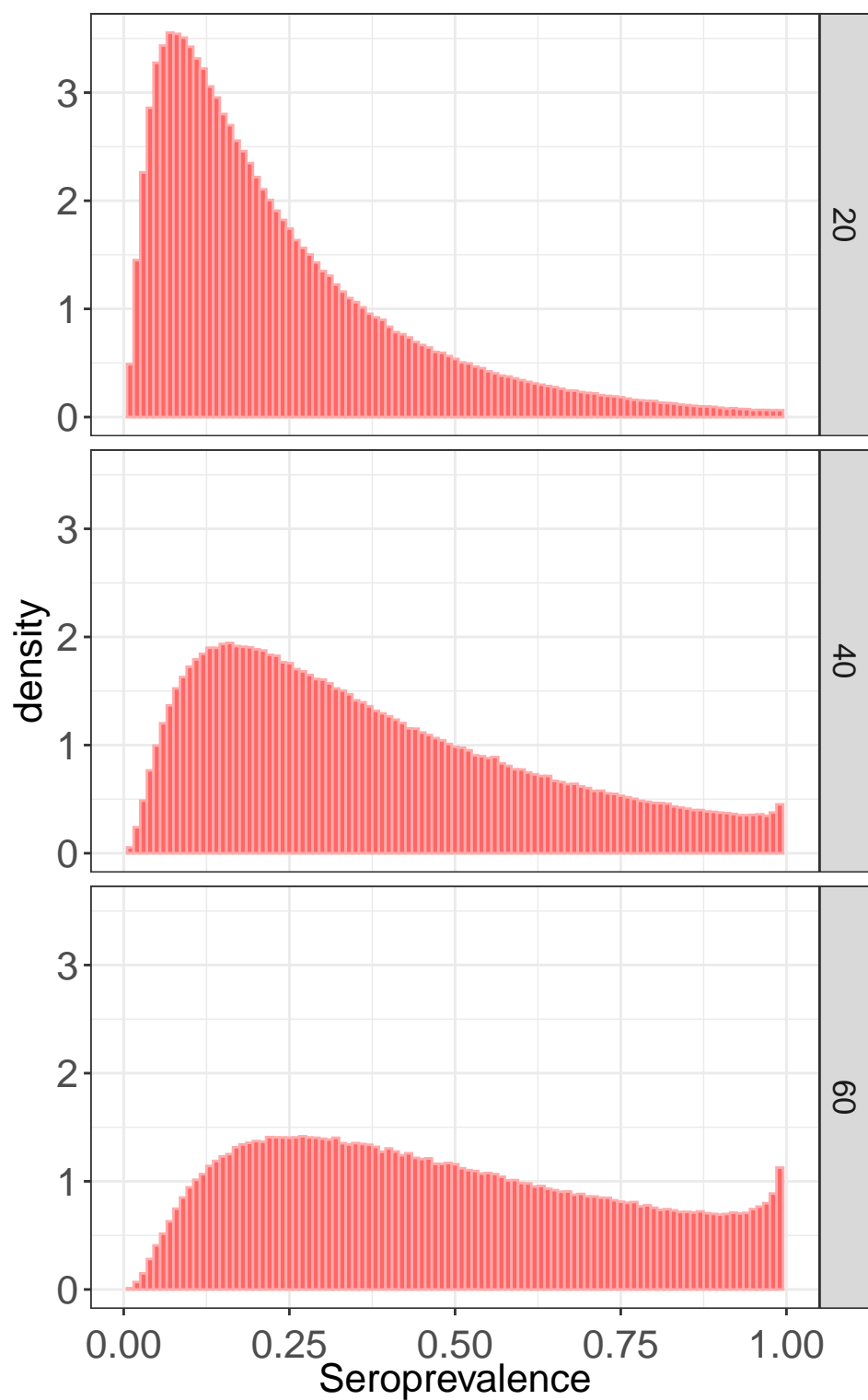

Supplement: S5 Fig — (PDF) [file pcbi.1012777.s005.pdf]

lambda1: 0.05, lambda2: 0.2

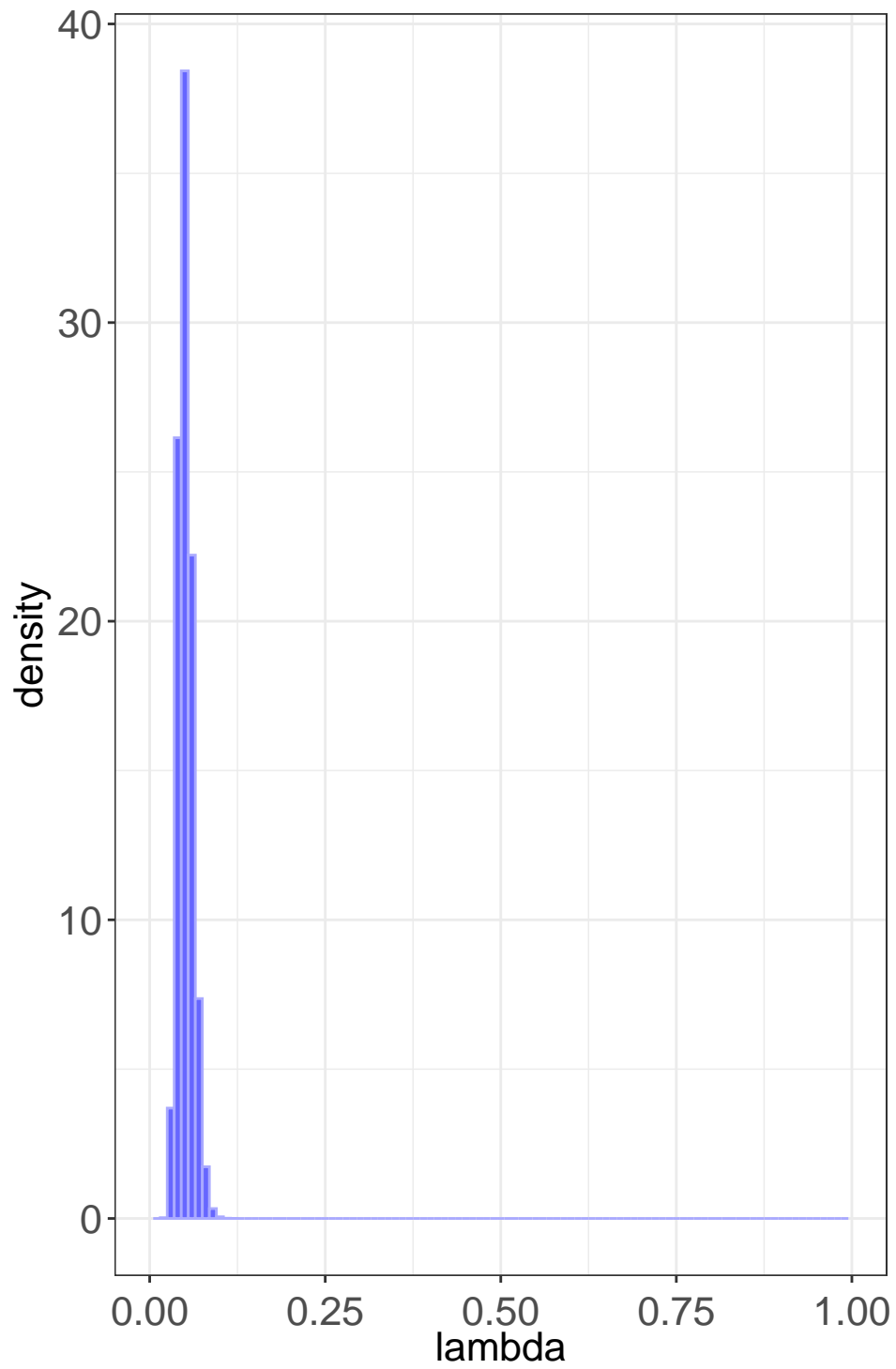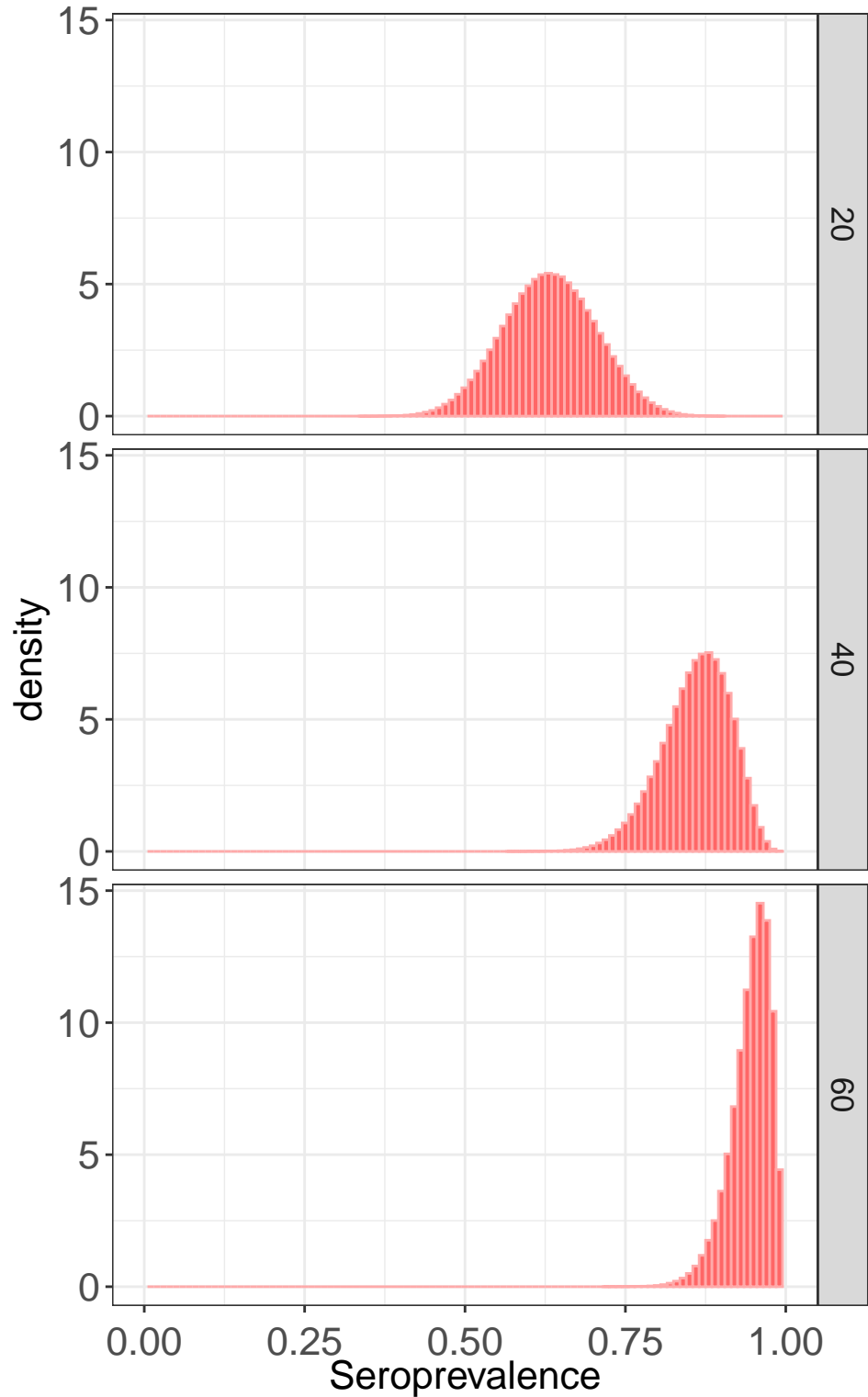

Supplement: S6 Fig — (PDF) [file pcbi.1012777.s006.pdf]

lambda1: 0.05, lambda2: 1

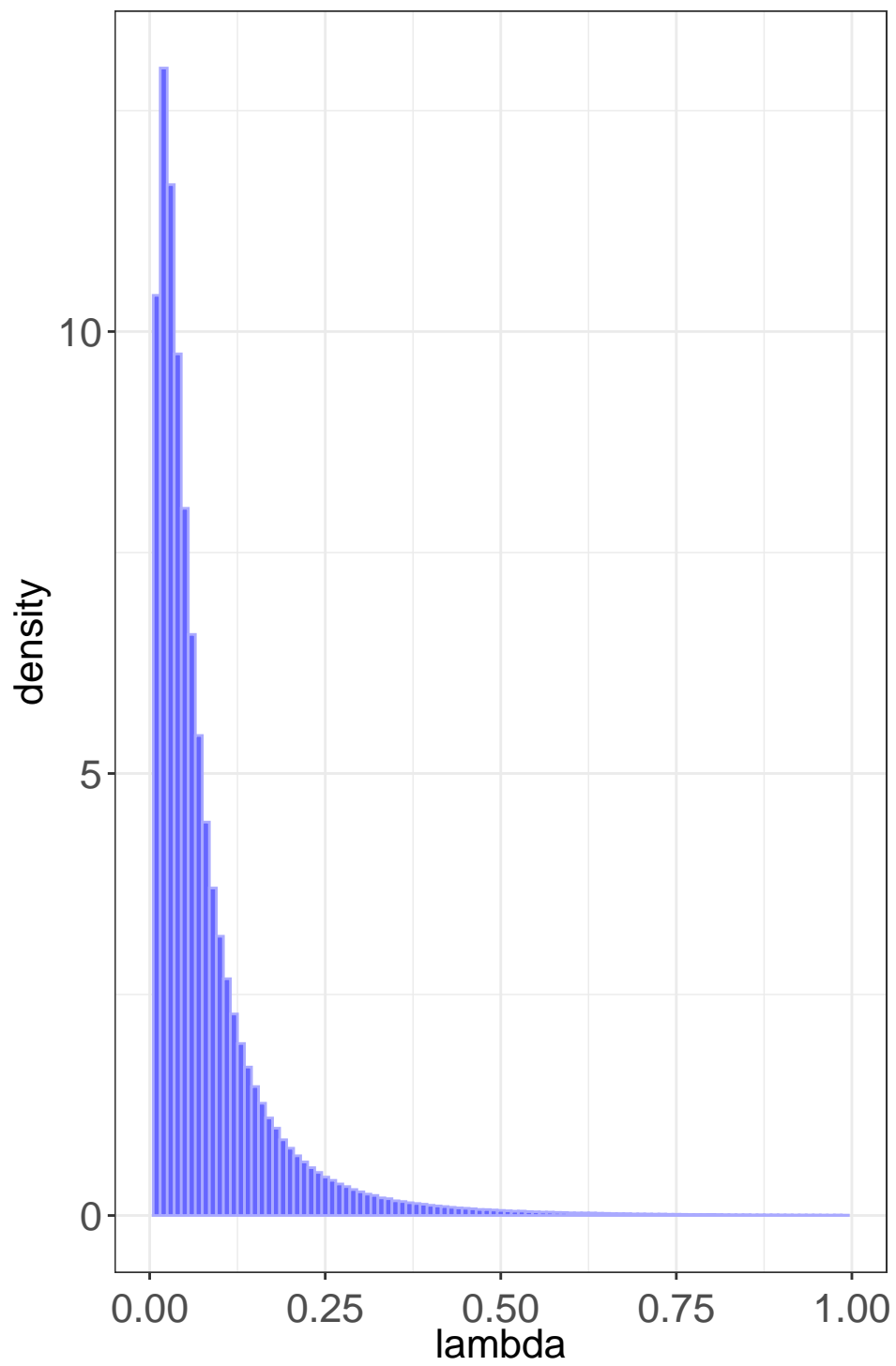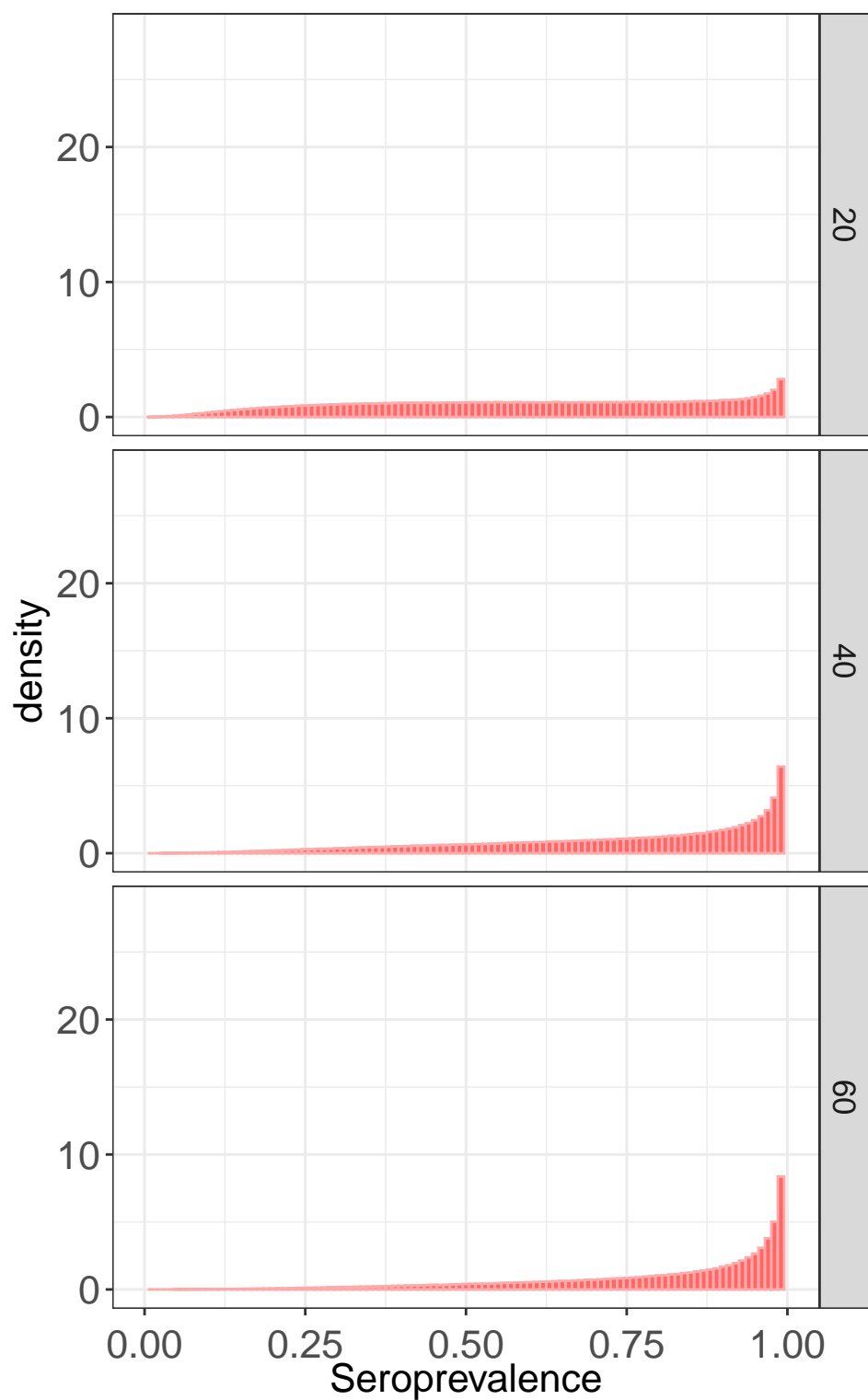

Supplement: S7 Fig — (PDF) [file pcbi.1012777.s007.pdf]
